# Fulminant Streptococcus suis Infection Causing Myocardial Damage and Toxic Shock-Like Syndrome: A Case Report

**DOI:** 10.7759/cureus.66625

**Published:** 2024-08-11

**Authors:** Taiki Hayasaka, Toshiyuki Ono, Toshiharu Takeuchi, Ayaka Sato, Yuta Kikuchi, Takahiro Kinebuchi, Mishie Tanino, Motoi Okada, Naoki Nakagawa

**Affiliations:** 1 Division of Cardiology and Nephrology, Department of Internal Medicine, Asahikawa Medical University, Asahikawa, JPN; 2 Department of Emergency Medicine, Asahikawa Medical University, Asahikawa, JPN; 3 Department of Clinical Laboratory, Social Welfare Corporation Hokkaido Social Work Association Furano Hospital, Furano, JPN; 4 Department of Diagnostic Pathology, Asahikawa Medical University Hospital, Asahikawa, JPN

**Keywords:** bacterial myocarditis, toxic shock-like syndrome, subendocardial biopsy, myocardial damage, streptococcus suis

## Abstract

There have been no prior reports of direct myocardial damage caused by *Streptococcus suis (S. suis)*, and understanding the clinical course of myocardial involvement is crucial for early diagnosis and initiation of treatment for this infection. A male pig farmer presented as an outpatient with a fever and sore throat, but within hours, his cardiac function declined, and his general condition deteriorated. Despite receiving comprehensive treatment, he succumbed to complications associated with toxic shock-like syndrome (TSLS). Blood cultures identified *S. suis*, and myocardial pathology revealed the presence of this bacterium in necrotic areas. This case marks the first reported instance of myocardial damage accompanied by TSLS due to *S. suis*, highlighting the significance of this infection.

## Introduction

*Streptococcus suis* is a pathogen that primarily causes meningitis, sepsis, and arthritis primarily in pigs worldwide. While *S. suis* is mainly a porcine pathogen, it also has zoonotic potential [1]. Although human cases are rare, their frequency has been increasing, particularly in regions with close human-swine contact, such as Southeast Asia, East Asia, and parts of Europe [1,2]. Human infection was first documented in Denmark in 1968, and the subsequent outbreaks especially in China and other countries have heightened concerns about its zoonotic transmission [3-6]. Humans typically come into direct contact with infected pigs or by consuming raw pork products [7]. The most common clinical manifestation of human infection is meningitis, followed by sepsis, arthritis, and the rare occurrence of endocarditis [8]. Notably, toxic shock-like syndrome (TSLS) resulting from *S. suis* infection has also been described, though it is rare and associated with a high mortality rate [9]. To date, however, myocarditis or direct bacterial involvement in humans has not been reported.

## Case presentation

A 61-year-old man, who owned and operated a pig farming business, presented to the outpatient clinic with a two-day history of generalized malaise, fever, and a sore throat. He had no significant previous medical history. On initial examination, his vital signs were as follows: blood pressure 124/68 mmHg, pulse rate 84/min, respiratory rate 20/min, and body temperature 38.2°C. Physical examination revealed mild bilateral pharyngeal erythema without exudates or cervical lymphadenopathy. With no organ-specific findings noted, acute pharyngitis was diagnosed and acetaminophen was prescribed.

After returning home, the patient developed epigastric pain, nausea, and vomiting. Approximately five hours later, the patient had repeated brief episodes of loss of consciousness, which prompted an emergency transfer. When the patient came to the hospital, his blood pressure was 60/30 mmHg and he was in a mild state of confusion. In addition, cold extremities, sweating, and reticular rash were observed. Echocardiography revealed severe diffuse left ventricular hypo-contractility. However, no pleural effusion was noted. Since acute fulminant myocarditis was suspected, the patient was transferred to the critical care center.

**Figure 1:**
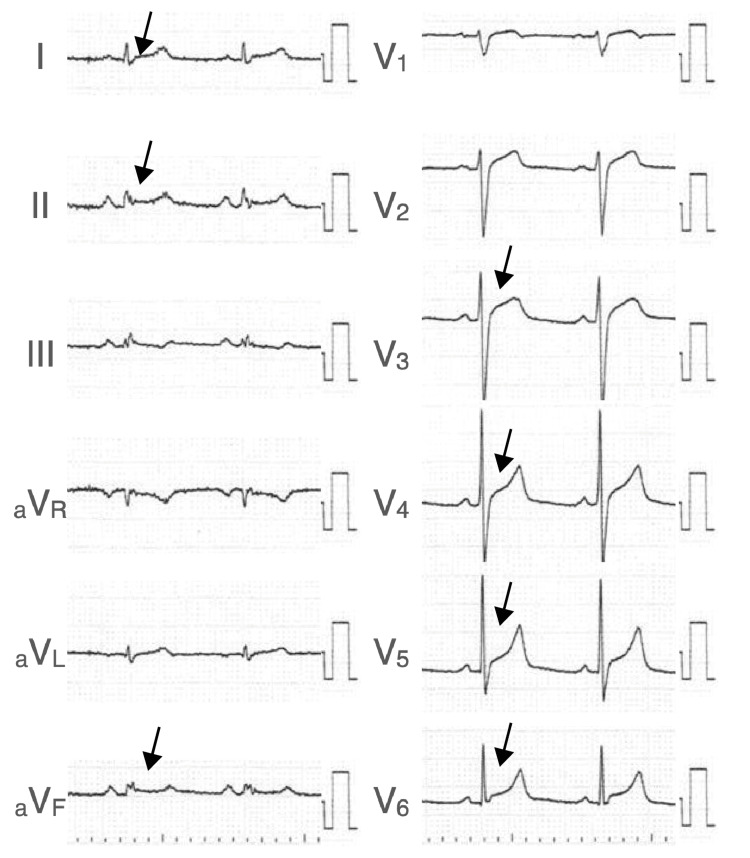
Transthoracic electrocardiogram obtained upon admission to the tertiary care center Heart rate was 75 bpm in sinus rhythm, and there was mild ST-segment elevation in the wide range of leads including I, II, aVF, and V3 to V6.

After transfer, the patient presented with mixed acid-base disturbances, including lactic acidosis, hyperbilirubinemia, hypoglycemia, and elevated procalcitonin (Table 1). Prompt administration of 50% glucose solution was initiated to address the hypoglycemia. Despite successful normalization of serum glucose levels, the patient's altered mental status remained unchanged. The electrocardiogram displayed low-voltage in the extremity leads and widespread non-specific mild ST-segment elevations (Figure 1). Echocardiography confirmed reduced wall motion of the entire left ventricle with an ejection fraction of 15%.

**Table 1:** Laboratory Findings on Admission

Parameters		Reference range (units)
White Blood Cell	1040	3300–8600 (/μL)
Red Blood Cell	430	435–555 (x10^4^/μL)
Hemoglobin	13.3	13.7–16.8 (g/dL)
Hematocrit	41.8	40.7–50.1 (%)
Platelet	2.9	15.8–34.8 (x10^4^/μL)
Prothrombin Time–International Normalized Ratio	>8.0	0.8–1.2
Activated Partial Thromboplastin Time	149.6	27.0–39.9 (sec)
D-dimer	64.4	0.00–0.50 (μg/mL)
Fibrinogen Degradation Products	175.5	0.0–9.9 (μg/mL)
Antithrombin-Ⅲ	52	83–115 (%)
Fibrinogen	50	160–350 (mg/dL)
Total Protein	5	6.6–8.1 (g/dL)
Albumin	2.5	4.1–5.1 (g/dL)
Total Bilirubin	2.8	0.4–1.5 (mg/dL)
Direct Bilirubin	1.3	0.0–0.2 (mg/dL)
Alkaline Phosphatase	122	38–113 (U/L)
Aspartate Aminotransferase	506	13–30 (U/L)
Alanine Aminotransferase	277	10–42 (U/L)
Lactate Dehydrogenase	887	124–222 (U/L)
Amylase	211	44–132 (U/L)
Creatine Kinase	240	59–248 (U/L)
Creatine Kinase-MB	10	0–12 (U/L)
Troponin I	13	0.0–26.2 (pg/mL)
Blood Urea Nitrogen	25.8	8.0–20.0 (mg/dL)
Creatinine	2.4	0.65–1.07 (mg/dL)
Uric Acid	8	0.0–7.0 (mg/dL)
Sodium	138	138–145 (mmol/L)
Potassium	4.9	3.6–4.8 (mmol/L)
Chloride	105	101–108 (mmol/L)
Glucose	3	73–109 (mg/dL)
C-Reactive Protein	5.4	0.00–0.14 (mg/dL)
Procalcitonin	53.49	0.00–0.49 (ng/mL)
N-Terminal Pro-Brain Natriuretic Peptide	1306	0.00–124.99 (pg/mL)
Arterial blood gas analysis		
(on 10 L/min of O_2_ via non-rebreathing mask)		
pH	7.096	7.35–7.45
Partial Pressure of Carbon Dioxide	18	35–45 (mmHg)
Partial Pressure of Oxygen	97.1	90–100 (mmHg)
Bicarbonate	8.3	20–26 (mmol/L)
Lactate	134	5–12 (mg/dL)
Base Excess	-23.8	-3.3–+2.3 (mEq/L)
Anion Gap	24.7	12–16 (mEq/L)

**Figure 2:**
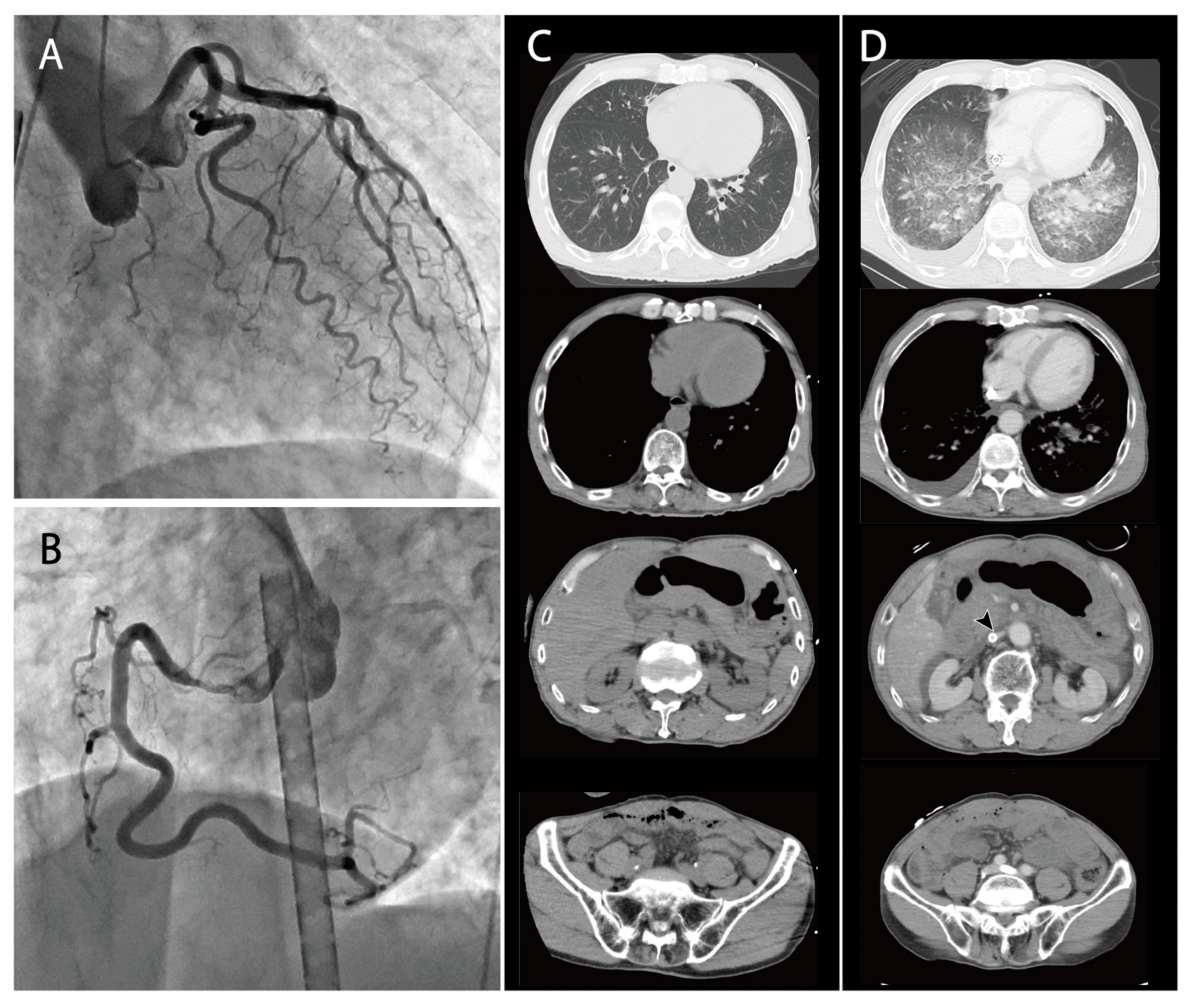
Diagnostic Imaging Results (A, B) Coronary angiogram of the left coronary artery (A) and the right coronary artery (B) revealed no significant stenosis. (C) Computed tomography (CT) obtained at the referring hospital did not show any evidence of chronic heart disease or infection foci. (D) Post-extracorporeal membrane oxygenation (ECMO) initiation contrast-enhanced CT scan demonstrated thickening of the interlobular septa (arrowhead), findings consistent with alveolar pulmonary edema, as well as the presence of pleural and ascitic fluid. The ECMO cannula was inserted into the inferior vena cava, which was collapsed.

He subsequently developed bradycardia and went into cardiopulmonary arrest. Cardiopulmonary resuscitation was performed, and venous-arterial extracorporeal membrane oxygenation (V-A ECMO) therapy was initiated. Coronary angiography excluded the possibility of acute myocardial infarction (Figures 2A, 2B), and a right ventricular endomyocardial biopsy was performed.

Initial computed tomography (CT) imaging showed no signs of cardiac enlargement or cardiomegaly indicative of chronic ventricular failure, no pericardial effusion, and no evidence of infection in other organs (Figure 2C). Due to the rapid deterioration of the patient's overall condition and the need to confirm proper ECMO cannula placement, a repeat CT scan was performed three hours later. This follow-up imaging revealed new findings of pulmonary edema, bilateral pleural effusions, and ascites. The inferior vena cava was severely collapsed despite the large volume of fluid administered. The ECMO cannulas were confirmed to be properly positioned. No space-occupying lesions or biliary obstructions that could cause liver dysfunction were observed (Figure 2D).

**Figure 3:**
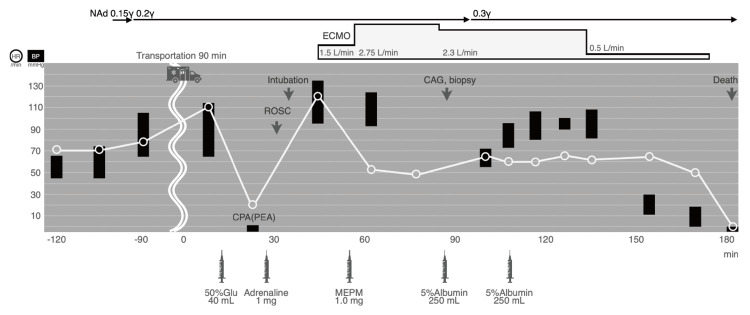
Clinical Course and Intervention Outcomes The patient was transferred while receiving a continuous infusion of norepinephrine. Immediately after arrival, he developed sudden bradycardia leading to pulseless electrical activity (PEA), prompting the initiation of cardiopulmonary resuscitation (CPR). Following the administration of epinephrine, the patient's heartbeat was restored. In the emergency department, the patient was intubated and placed on mechanical ventilation, and extracorporeal membrane oxygenation (ECMO) was established. A myocardial biopsy was also performed. However, despite the administration of large volumes of crystalloid fluids and albumin, poor venous drainage through the ECMO circuit and progressive abdominal distension occurred. The patient's condition rapidly deteriorated, and he ultimately succumbed to the illness three hours after arrival, despite resuscitative efforts. γ: μg/kg/min, NAd: Norepinephrine, ECMO: Extracorporeal membrane oxygenation, HR: Heart rate, BP: Blood pressure, CPA: Cardiopulmonary arrest, PEA: Pulseless electrical activity, ROSC: Return of spontaneous circulation, CAG: Coronary angiography, ICU: Intensive care unit, Glu: Glucose, MEPM: Meropenem

The patient was considered to be in septic shock, and meropenem was started along with aggressive fluid resuscitation and norepinephrine. Unfortunately, despite multidisciplinary treatment, his condition rapidly deteriorated. Despite ongoing fluid resuscitation, the patient was unable to maintain adequate circulating plasma volume, leading to progressive circulatory failure. This ultimately resulted in the patient's death, despite continuous resuscitative efforts (Figure 3).

**Figure 4:**
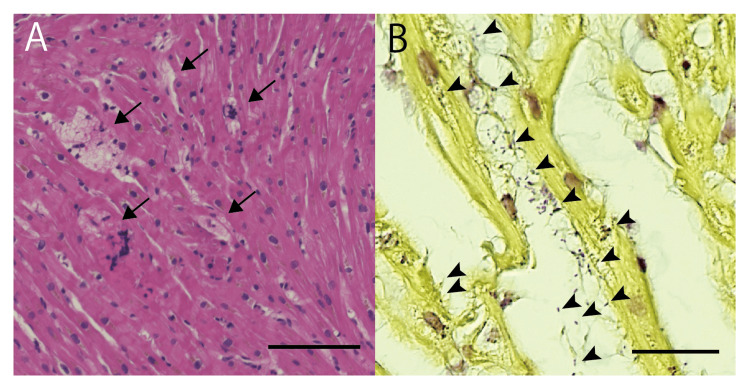
Myocardial Tissue Histopathology (A) Hematoxylin and eosin (H&E) staining shows scattered myocardial cell necrosis, vacuolar degeneration and dropout (arrow). However, there is no inflammatory cell infiltration. Scale bar = 100 μm. (B) Gram staining reveals Gram-positive cocci forming pairs or short chains (arrowheads) localized to the areas of myocardial necrosis. Scale bar = 25 μm.

Bacteria were detected in blood culture and exhibited alpha hemolysis on sheep blood agar. The serotype was identified as type 2, and biochemical testing using RAPID ID 32 STREP (BIOMÉRIEUX) indicated that the organism was *S.suis*. Mass spectrometry with the MALDI Biotyper (BRUKER) and 16SrRNA gene analysis further confirmed that the bacterium was indeed *S. suis*. Myocardial biopsy pathology revealed scattered areas of myocardial cell necrosis with fibrin deposition and a chain of Gram-positive cocci consistent with the site of myocardial injury (Figure 4). The clinical course was consistent with TSLS due to *S. suis* infection, and we hypothesized that the severe cardiac dysfunction was due to direct bacterial infiltration and necrosis of the myocardium.

## Discussion

This case describes a rare case of *S. suis* infection causing TSLS with severe myocardial involvement. In this patient, rapid hemodynamic deterioration led to cardiogenic shock, and a comprehensive treatment approach included the initiation of VA-ECMO. Coronary angiography excluded acute coronary artery disease as the underlying cause of the cardiogenic shock.

*S. suis* is a Gram-positive streptococcus that often exhibits short chain or diplococci. It commonly displays alpha-hemolysis on sheep blood agar medium, and it has at least 29 known serotypes [10]. In the present case, serotype 2, which has been reported as the most common and pathogenic cause of human infections, was identified [11].

The primary routes of human infection are generally through wounds on the hands of pig farmers, butchers, or food service workers who are in close contact with pigs [3,7]. Indeed, the patient in this case was a pig farmer. On the other hand, cases with unknown bacterial entry points have been reported [3,7]. In this case, the patient had a preceding pharyngeal infection, which could suggest that a viral infection might have impacted the immune system and potentially facilitated the entry of *S. suis* into the body. Recently, infections have also been linked to the consumption of raw pork in Southeast Asia [7]. The most common clinical presentation is meningitis; however, sepsis and arthritis have also been reported [8]. While rare cases of TSLS have been documented, myocarditis or other cardiac lesions caused by S. suis infection have not been reported. To investigate this topic, a comprehensive search was conducted on PubMed using relevant keywords, but no additional cases of myocarditis or cardiac lesions associated with *S. suis* infection were found.

This case highlights two important observations regarding *S. suis* infection. First, *S. suis* can invade the myocardium and directly cause myocardial damage. Echocardiography revealed markedly impaired overall cardiac function and endomyocardial biopsy confirmed the presence of bacteria in close proximity to necrotic myocardial cells. Although attempts were made to identify the bacteria in the myocardial tissue, the biopsy sample was too small for genetic analysis, and culture from formalin-fixed tissue was unsuccessful. However, the morphology of the bacteria observed by Gram staining was consistent with *S. suis*, which was also grown in blood culture, suggesting that the myocardial damage was directly caused by this pathogen.

Nevertheless, this condition differs from typical myocarditis in several aspects. One key difference is the lack of observed elevation in cardiac enzymes such as troponin in the serum. This may be due to the limited extent of myocardial necrosis and the dilution effect from the massive fluid resuscitation required. Another distinction is the absence of significant inflammatory cell infiltrate in the myocardial tissue. Thus, this condition appears not to be typical myocarditis but rather a sepsis-related myocardial injury. Additionally, while decreased myocardial contractility can occur in septic cardiomyopathy, the presence of bacteria within the myocardial tissue distinguishes this case from typical septic cardiomyopathy.

On the other hand, when *S. suis* infection causes structural changes in organs such as the liver and spleen, inflammatory cell infiltration has been reported to be relatively mild [9]. The thick capsular polysaccharide of *S. suis* helps the bacteria evade the innate immune system and promotes tissue infiltration [12,13]. In summary, although the bacteria present in the myocardium caused dysfunction through myocardial necrosis and toxin production, there was no significant inflammatory cell infiltration in the acute phase. The absence of a conspicuous inflammatory cell infiltrate is thought to be due to the bacteria’s ability to evade the innate immune response.

The second important point is that the clinical course was extremely intense. Despite early V-A ECMO to support compromised cardiac function, capillary permeability increased due to TSLS. Plasma continuously leaked into the abdominal and thoracic cavities, did not respond to treatment with large volumes of intravenous fluids and albumin administration, and the patient was unable to maintain circulating blood volume, leading to death. The mortality rate for *S. suis* infections is reported to be about 12%, but the majority of deaths are due to sepsis, including TSLS [14]. In this case, the decreased circulating plasma volume, and the reduction in cardiac output both combined to cause hemodynamic compromise and multiple organ failure, which was not life-saving.

Initially, the patient presented to the outpatient clinic with fever and mild pharyngitis, which was treated as a flu-like illness without a specific diagnosis. However, had his occupation as a pig farmer been carefully interviewed, the possibility of this infection could have been considered earlier. Currently, growing concern about antibiotic resistance in *S. suis* has raised hopes for vaccine development [15]. Nevertheless, given the unique bacterial characteristics of this pathogen, human vaccination has not yet been implemented [16].

Raising awareness of high-risk populations, such as pig farmers, butchers, those with occupational contact with pigs, and populations with cultural practices of consuming raw pork, remains critically important as a preventive measure against *S. suis* infections. In a severe infection such as this one, prompt diagnosis and appropriate initiation of treatment are key to successful outcomes. Through this case report, we hope to increase awareness of this potentially life-threatening infection among those at risk for pork exposure.

## Conclusions

This case report highlights the critical implications of *S*.* suis* infections, emphasizing the potential for severe myocardial damage and rapid clinical deterioration. It demonstrates *S. suis*'s capability to directly invade and damage the myocardium while simultaneously inducing TSLS, leading to a fulminant course. The rapid progression from initial symptoms to severe cardiac dysfunction underscores the importance of early recognition and prompt intervention, particularly in high-risk populations such as pig farmers. In this case, pathological and microbiological approaches were necessary to reach an accurate diagnosis. Moving forward, this case emphasizes the importance of considering *S. suis* in the differential diagnosis of rapidly progressing infections with cardiac involvement. Timely diagnosis and appropriate treatment remain essential for managing severe *S. suis* infections.

## References

[1] Lun ZR, Wang QP, Chen XG, Li AX, Zhu XQ (2007). Streptococcus suis: an emerging zoonotic pathogen. Lancet Infect Dis.

[2] Murray GG, Hossain AS, Miller EL (2023). The emergence and diversification of a zoonotic pathogen from within the microbiota of intensively farmed pigs. Proc Natl Acad Sci U S A.

[3] Yu H, Jing H, Chen Z (2006). Human Streptococcus suis outbreak, Sichuan, China. Emerg Infect Dis.

[4] Praphasiri P, Owusu JT, Thammathitiwat S (2015). Streptococcus suis infection in hospitalized patients, Nakhon Phanom Province, Thailand. Emerg Infect Dis.

[5] Brizuela J, Kajeekul R, Roodsant TJ (2023). Streptococcus suis outbreak caused by an emerging zoonotic strain with acquired multi-drug resistance in Thailand. Microb Genom.

[6] Perch B, Kristjansen P, Skadhauge K (1968). Group R streptococci pathogenic for man. Two cases of meningitis and one fatal case of sepsis. Acta Pathol Microbiol Scand.

[7] Rayanakorn A, Goh BH, Lee LH, Khan TM, Saokaew S (2018). Risk factors for Streptococcus suis infection: a systematic review and meta-analysis. Sci Rep.

[8] Huong VT, Ha N, Huy NT (2014). Epidemiology, clinical manifestations, and outcomes of Streptococcus suis infection in humans. Emerg Infect Dis.

[9] Tang J, Wang C, Feng Y (2006). Streptococcal toxic shock syndrome caused by Streptococcus suis serotype 2. PLoS Med.

[10] Segura M, Aragon V, Brockmeier SL (2020). Update on Streptococcus suis research and prevention in the era of antimicrobial restriction: 4th International Workshop on S. suis. Pathogens.

[11] Gottschalk M, Segura M, Xu J (2007). Streptococcus suis infections in humans: the Chinese experience and the situation in North America. Anim Health Res Rev.

[12] Deng S, Liao J, Li H (2024). Streptococcus suis subtilisin-like serine proteases SspA-1 and SspA-2 interplay with complement C3a and C5a to facilitate bacterial immune evasion and infection. Virulence.

[13] Li Z, Chang P, Xu J, Tan C, Wang X, Bei W, Li J (2019). A Streptococcus suis live vaccine suppresses Streptococcal toxic shock-like syndrome and provides sequence type-independent protection. J Infect Dis.

[14] Rayanakorn A, Katip W, Goh BH, Oberdorfer P, Lee LH (2019). Clinical manifestations and risk factors of Streptococcus suis mortality among Northern Thai population: retrospective 13-year cohort study. Infect Drug Resist.

[15] Costinar L, Badea C, Marcu A, Pascu C, Herman V (2024). Multiple drug resistant Streptococcus strains-an actual problem in pig farms in Western Romania. Antibiotics (Basel).

[16] Segura M (2015). Streptococcus suis vaccines: candidate antigens and progress. Expert Rev Vaccines.

